# Oncostatin M Maintains the Hematopoietic Microenvironment in the Bone Marrow by Modulating Adipogenesis and Osteogenesis

**DOI:** 10.1371/journal.pone.0116209

**Published:** 2014-12-31

**Authors:** Fumi Sato, Yuichiro Miyaoka, Atsushi Miyajima, Minoru Tanaka

**Affiliations:** 1 Laboratory of Cell Growth and Differentiation, Institute of Molecular and Cellular Biosciences, The University of Tokyo, Tokyo, Japan; 2 Laboratory of Stem Cell Regulation, Institute of Molecular and Cellular Biosciences, The University of Tokyo, Tokyo, Japan; 3 Department of Regenerative Medicine, Research Institute, National Center for Global Health and Medicine, Tokyo, Japan; Toho University School of Medicine, Japan

## Abstract

The bone marrow (BM) is an essential organ for hematopoiesis in adult, in which proliferation and differentiation of hematopoietic stem/progenitor cells (HSPC) is orchestrated by various stromal cells. Alterations of BM hematopoietic environment lead to various hematopoietic disorders as exemplified by the linking of fatty marrow with increased adipogenesis to anemia or pancytopenia. Therefore, the composition of mesenchymal stromal cell (MSC)-derived cells in the BM could be crucial for proper hematopoiesis, but the mechanisms underlying the MSC differentiation for hematopoiesis remain poorly understood. In this study, we show that Oncostatin M (OSM) knock out mice exhibited pancytopenia advancing fatty marrow with age. OSM strongly inhibited adipogenesis from BM MSC *in vitro*, whereas it enhanced their osteogenesis but suppressed the terminal differentiation. Intriguingly, OSM allowed the MSC-derived cells to support the *ex vivo* expansion of HSPC effectively as feeder cells. Furthermore, the administration of OSM in lethally irradiated wild-type mice blocked fatty marrow and enhanced the recovery of HSPC number in the BM and peripheral blood cells after engraftment of HSPC. Collectively, OSM plays multiple critical roles in the maintenance and development of the hematopoietic microenvironment in the BM at a steady state as well as after injury.

## Introduction

The bone marrow (BM) is a major tissue that supplies blood throughout life. Hematopoietic stem cells (HSC) are surrounded by various types of stromal cells and the proliferation and differentiation of HSC is tightly regulated in the BM microenvironment [1]. Two types of functional niches for supporting HSC in the BM have been studied; i.e., the osteoblastic niche [2]–[4] and perivascular niche [5]–[7], which are composed of osteoblasts and endothelial cells/perivascular mesenchymal cells, respectively. Mesenchymal stromal cells (MSC) in the BM can give rise to multiple cell lineages *in vitro*, including adipocytes, osteocytes, and chondrocytes [8], [9]. Recently, it has been reported that a subset of MSC (PDGFα+Sca-1+CD45-TER119-; PαS cell) in the BM could differentiate into hematopoietic niche cells, osteoblasts, and adipocytes after *in vivo* transplantation [10], although it remains to be elucidated whether the PαS-derived cells function as HSPC niche in the BM and what factors regulate the differentiation of PαS cell *in vivo*.

On the other hand, Naveiras et al. reported the BM adipocytes as negative regulators of the hematopoietic microenvironment using lipoatrophic A-ZIP/F1 ‘fatless’ mice [11]. In the study, transplantation of normal BM cells in lethally irradiated A-ZIP/F1 mice lacking adipogenesis showed enhanced hematopoietic recovery compared with wild-type recipient mice, indicating that adipocytes in fatty marrow hinder hematopoietic progenitor expansion. In humans, it is also known that fatty marrow gradually predominates with age [12]. It should be noted that some myeloid diseases such as aplastic anemia (AA) displaying anemia, and/or pancytopenia are accompanied by severe fatty marrow [13]. These reports strongly suggest that the cellularity of adipocytes, osteoblasts and other mesenchymal stromal cells in the BM is critical for an adequate hematopoietic microenvironment. As these cells could be derived from MSC in the BM, the factor(s) that regulate their balance would be a novel therapeutic target for impaired hematopoiesis in fatty marrow and for enhancing hematopoietic engraftment after BM transplantation.

OSM is a member of the interleukin (IL)-6 family of cytokines and has various unique biological activities e.g. hematopoiesis, hepatogenesis and adipogenesis, which are not shared by the other family members [14]. Murine OSM is expressed in the aorta-gonad-mesonephros (AGM) region, where long-term repopulating HSC (LT-HSC) arise, and OSM stimulates the expansion of multipotential hematopoietic progenitors in the primary culture of AGM [15]. While OSM also expanded hematopoietic stem/progenitor cells (HSPC) in co-cultures of AGM and fetal liver cells [16], it induced the differentiation of fetal hepatocytes *in vitro* [17]. Despite its strong and unique activities *in vitro*, the genetic ablation of either OSM or its receptor (OSMR) in mice unexpectedly showed no severe defects during development [18], [19]. In contrast, these adult knockout (KO) mice displayed anemic and thrombocytopenic phenotype although the symptoms were relatively mild. Colony-forming unit (CFU) assays showed that the number of hematopoietic progenitors in BM was significantly reduced in KO mice compared to wild-type (WT) mice, whereas that in spleen was increased. Additionally, the number of hematopoietic progenitors in peripheral blood was increased in OSM KO mice, indicating the mobilization of HSPC from the BM into the circulation. These reports suggest the possibility that the BM niche harboring HSPC is impaired in OSM KO mice.

OSM was shown to inhibit the adipocytic differentiation of 3T3L1 cells, a preadipocyte cell line, mouse embryonic fibroblasts (MEFs), adipose tissue-derived MSC and BM MSC [20]–[22]. Considering the BM adipocytes as a negative regulator of the hematopoietic microenvironment [11], the lack of inhibitory effect of OSM on adipogenesis from MSC may account for the reduced hematopoietic activity in OSM KO BM. However, the causal linkage between these two original findings has remained uninvestigated. Although Walker et al. reported that marrow adipocyte volume was increased in 12-week-old OSMR KO mice [22], the role of OSM in the BM microenvironment for hematopoiesis has not been elucidated. By contrast, several reports have demonstrated that OSM induces osteogenic differentiation of human MSC and a murine stromal cell line [21]–[23], suggesting a role for OSM in the hematopoietic microenvironment.

In this report, we aimed to unravel the roles of OSM in the BM hematopoietic microenvironment under various conditions, BM injuries by chemicals or irradiation and aging. In addition, we prepared PαS MSC from WT and OSMR KO BM to examine the biological activities of OSM in their adipogenic and osteogenic differentiation and evaluated the hematopoietic capacity by developing a co-culture system with HSPC. We further present the possibility to use OSM as a promising therapeutic agent to enhance the recovery of hematopoiesis after BM lesion. Our results demonstrate that OSM contributes to the maintenance of the hematopoietic microenvironment by balancing multiple steps of differentiation of BM MSC in both steady and injured states.

## Materials and Methods

### Mice

C57BL/6J mice were purchased from Clea Japan, Inc. (Tokyo, Japan). CD45.1 (C57BL/6) congenic mice were also purchased from the RIKEN Research Center. OSM KO mice and OSMR KO mice were generated as described previously [18], [19]. All animals were maintained in a standard Specific-Pathogen Free (SPF) room at our animal facility. All animal experiments were performed according to the guidelines approved by the Institutional Animal Care and Use Committee of the University of Tokyo.

### Surgical and chemical treatment

For splenectomy, the spleen was removed surgically from anesthetized 10-week-old mice after the splenic artery was ligated by 4-0″ suture (Vicryl; Ethicon, Inc., Somerville, NJ, USA). For myeloablation, 10 mg of busulfan (Sigma-Aldrich, St. Louis, MO, USA) were dissolved in 1 mL of acetone (Wako Chemical Co., Kyoto, Japan), and then 4 mL of sterilized distilled water were added to yield a final concentration of 2 mg/mL. Next, 20 mg/kg of busulfan was administered intraperitoneally three times over a 1-week period. Mice were sacrificed 1 week after the final administration.

### Measurement of hematocrit values and plasma Erythropoietin (EPO) levels

Peripheral blood was placed into heparinized Micro-Hematocrit Capillary tubes (Thermo Fisher Scientific, Waltham, MA, USA) and centrifuged at 3000 rpm for 20 min at room temperature. After determining the hematocrit value, the plasma was collected for measurement of the plasma EPO concentration. The plasma EPO concentration was measured using a mouse EPO ELISA kit as described in the manufacturer's protocol (R&D Systems, Minneapolis, MN, USA).

### Preparation of BM sections

The femurs were excited and immersion-fixed in 10% neutral-buffered formalin, pH 7.0–7.5 (Wako) at 4°C for 24 h. After washing under gently running water, the femurs were demineralized at 4°C for 12 days in decalcifying Solution B (0.5 M EDTA, pH 7.4) (Wako). For Oil Red O staining, demineralized femurs were immersed sequentially in 5, 10, 15, and 20% sucrose-phosphate-buffered saline (PBS) for 6 h each. Finally, the femurs were embedded in OCT compound (Sakura Finetek Inc., Torrance, CA, USA) and sliced into 14-µm sections using a cryostat (MICROM HM 525; Thermo Fisher Scientific).

### Isolation of PαS cells from adult mouse BM

PαS cells, which co-expressed PDGFRα and Sca-1, but not CD45 and TER119, were isolated from the bone of WT or OSMR KO mice as described by Morikawa et al. [10]. Briefly, bone marrow was flushed out from femurs and tibias of 8–12 mice using 22- and 23-G needles (Terumo) and the remaining bone was ground with a pestle. Then, the crushed bone fragments were treated with 0.2% collagenase (Sigma) for 30 min at 37°C. Next, after depletion of mature red blood cells using hypotonic lysis buffer (0.38% NH_4_Cl) for 7 min on ice, the cells were incubated with anti-FcR for 15 min on ice. The cells were then incubated with allophycocyanin (APC)-conjugated rat anti-mouse PDGFRα (APA5; eBioscience, San Diego, CA, USA), fluorescein isothiocyanate (FITC)-conjugated rat anti-mouse Sca-1 (E13-161.7; BD Biosciences, Franklin Lakes, NJ, USA), phycoerythrin (PE)-conjugated rat anti-mouse CD45 (30-F11; BD Biosciences), and PE-conjugated rat anti-mouse TER119 (TER-119; eBioscience). After dead cells were excluded by propidium iodide staining, the CD45^-^ TER119^-^ PDGFRα^+^ Sca-1^+^ cells were sorted using a Moflo XDP cell sorter (Beckman-Coulter, Fullerton, CA, USA). MSC were sub-cultured in α-MEM (Invitrogen, Carlsbad, CA, USA) supplemented with 10% fetal bovine serum (JRH Biosciences, Inc., Lenexa, KS, USA), penicillin-streptomycin-glutamine solution (Invitrogen), and Gluta-Max (Invitrogen).

### Adipogenic differentiation assay

Adipocyte differentiation of PαS cells was performed using Adipogenic Induction Medium/Adipogenic Maintenance Medium (Lonza, Walkersville, MD, USA) and supplements from the Adipogenic Induction/Adipogenic SingleQuots Kit (Lonza), according to the manufacturer's protocol. Briefly, PαS cells were seeded at 10,000/well into six-well plates (Corning Inc., Corning, NY USA) and allowed to reach sub-confluence. Next, PαS cells were cultured with three cycles of Adipogenic Induction Medium/Adipogenic Maintenance Medium. OSM was added to only the Adipogenic Induction Medium. Oil Red O staining was performed as described previously [20].

### Osteogenic differentiation assay

Osteogenic differentiation of the PαS cells was performed using Osteogenic Induction Medium (Lonza) and supplements from the Osteogenic SingleQuots Kit (Lonza), according to the manufacturer's protocol. Briefly, PαS cells were seeded at 30,000/well in maintenance medium and allowed to attach. Next, the medium was changed to the osteogenic induction medium in the presence or absence of OSM. Alizarin Red S staining was used to evaluate osteoblastic differentiation by detecting mineralized nodules. Alizarin Red S (Sigma) was dissolved in distilled water, and the pH was adjusted to pH 6.38 by the addition of 0.25% ammonium hydroxide. Cultured cells were washed with PBS and fixed in 95% ethanol for 10 min at room temperature. After washing with distilled water, the cells were incubated in Alizarin Red S solution for 10 min at room temperature. The cells were then gently washed with distilled water, 95% and 100% ethanol.

### Real-time RT-PCR analyses

Total RNA was collected from the BM, spleen, and liver of WT or OSM KO mice using TRIzol reagent, according to the manufacturer's instructions (Invitrogen). After treatment with DNase I (Invitrogen), total RNA was reverse-transcribed using the Prime Script RT Reagent Kit (TaKaRa, Shiga, Japan) and subjected to real-time PCR analyses using a Light Cycler 480 (Roche, Mannheim, Germany). For adipogenic and osteogenic differentiation assays, real-time RT-PCR was performed as above. Data were normalized to beta-actin expression. Primer sequences are shown in S1 Table.

### Preparation of LSK cells from adult mouse BM

Single-cell suspensions of whole BM cells were labeled by incubation on ice for 15 min using a cocktail of the following antibodies: PE-conjugated anti-mouse CD3ε (145-2C11; BD Biosciences), CD4 (L3T4; BD Biosciences), CD8α (53-6.7; BD Biosciences), B220 (RA3-6B2; BD Biosciences), Mac-1 (M1/70; BD Biosciences), Gr-1 (RB6-8C5; eBioscience), and Ter119 (TER-119, eBioscience); FITC-conjugated anti-mouse Sca-1 (E13-161.7; BD Biosciences); and APC-conjugated anti-mouse c-Kit (2B8; BD Biosciences). After cells were washed with PBS, they were incubated with anti-PE microbeads (Miltenyi Biotec, Bergisch Gladbach, Germany) at 4°C for 15 min. Lin^+^ cells (CD4^+^, CD8α^+^, B220^+^, Mac-1^+^, Gr-1^+^, Ter119^+^ cells) were removed by immunomagnetic selection using a MACS system (Miltenyi Biotec). These negatively selected cells were subjected to FACS using a Moflo XDP cell sorter (Beckman-Coulter) to prepare LSK cells.

### Co-culture system

The co-culture system was developed by modifying the HSC expansion system [24]. WT-PαS cells were freshly sorted by FACS and plated at 2000/well in a 96-well plate (Corning Inc.). After PαS cells were cultured in maintenance medium until sub-confluent growth, the medium was changed to osteogenic induction medium in the presence or absence of OSM. At day 7 of osteogenic induction, the medium was removed, rinsed, and replaced with 200 µl of HSC culture medium containing 5,000 LSK cells. The HSC culture medium consists of StemSpan SFEM medium (Stemcell Technologies, Inc., Vancouver, British Columbia, Canada) supplemented with 50× penicillin/streptomycin (Life Technologies, Carlsbad, CA, USA), 10 ng/mL recombinant mouse SCF (Pepro Tech, Inc., Rocky Hill, NJ, USA). After co-culture, cells were inoculated at 37°C under 4% O_2_ and 5% CO_2_ conditions for 7 days. One hundred microliters of medium were replaced with fresh HSC culture medium every 2–3 days. At day 7 of co-culture, after treatment with 0.25% trypsin-EDTA, nucleated cells were enumerated using a hemocytometer after staining with Turk's solution (Wako). Re-analyses of the LSK fraction in expanded cells were performed using the BD FACS Canto II system (BD Biosciences). Data were analyzed using the FlowJo software.

### Irradiation and OSM administration

CD45.2 recipient mice were exposed to lethal dose of radiation (M-150WE; Softex) and BM cells prepared from CD45.1 donor mice were injected intravenously. Primary administration of OSM was performed in conjunction with BM transplantation at a dose of 600 ng per a mouse. Then, OSM was administrated intraperitoneally twice (10 a.m. and 6 p.m.) a day for 7 days. Mice were sacrificed at day 7 after irradiation, and the BM of femurs was analyzed by Oil red O staining and real-time RT-PCR. Blood was collected from a tail vein every 7 days during the recovery process, and analyzed by using an automated counter pocH-100iV (Sysmex).

### Statistical methods

Differences between means were evaluated using a standard unpaired, one-way Student's t-test. Data were indicated as the means ± standard deviation (S.D.) or standard error of the mean (S.E.M.). A value of p<0.05 was taken to indicate statistical significance.

## Results

### Impaired hematopoietic activity of OSM-deficient BM

**Figure 1:**
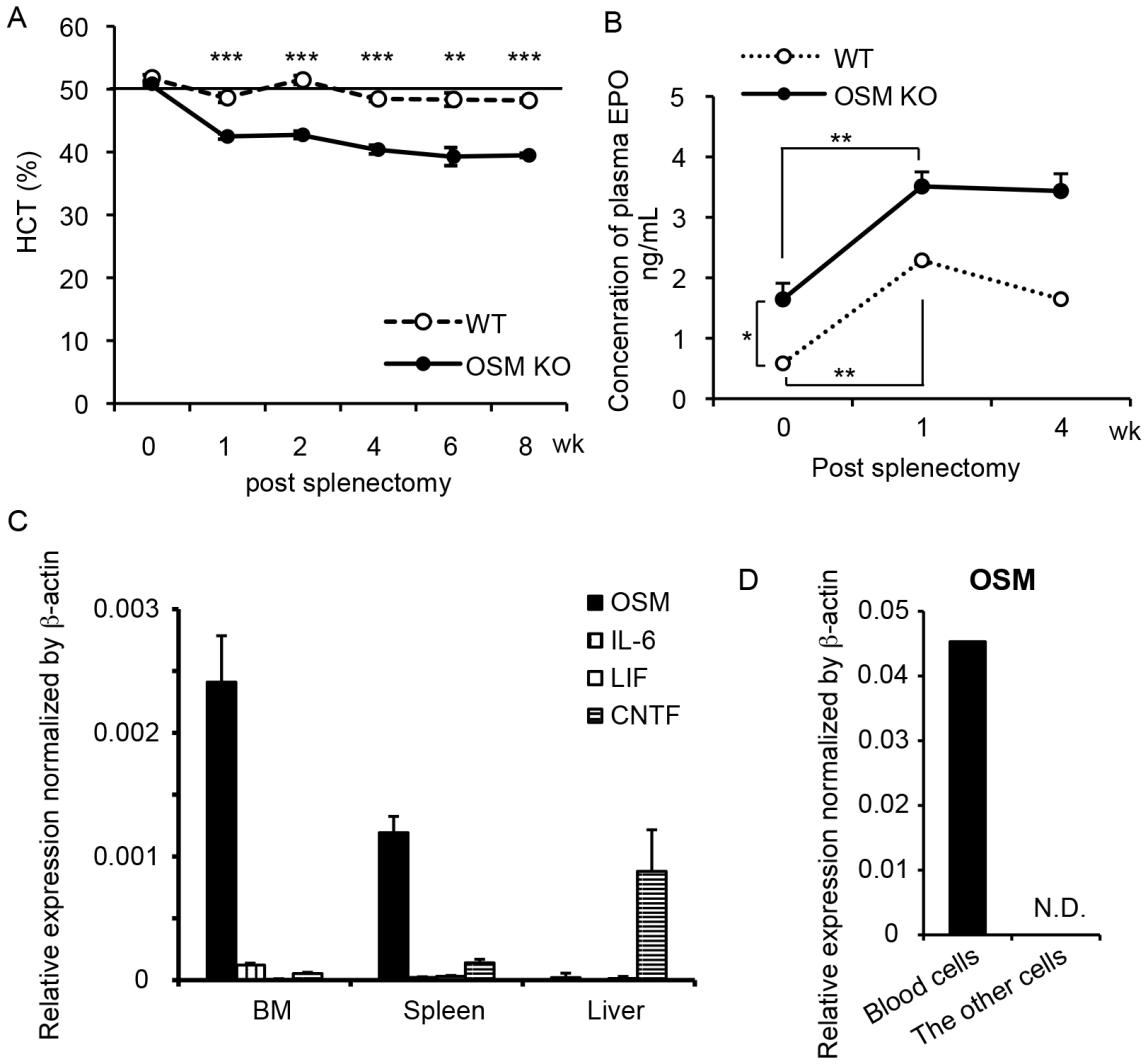
Impaired BM hematopoiesis in OSM KO mice. (A) Hematocrit values of WT (dashed line) and OSM KO mice (solid line) after Spx treatment. Hematocrit values in Spx-treated OSM KO mice showed no sign of recovery from anemic symptoms. Data are presented as means ± S.E.M. (n = 4). (B) Plasma concentrations of EPO in WT (dashed line) and OSM KO mice (solid line) by ELISA. Data are presented as means ± S.E.M. (n = 4). (C) Gene expression analysis of IL-6 family cytokines (OSM, IL-6, LIF, and CNTF) in the BM of WT mice. The expression level was normalized against that of β-actin. Data are presented as means ± S.D. (n = 4). (D) Real-time RT-PCR analysis of OSM mRNA expression in CD45^+^ Ter119^+^ cells (blood cells) and CD45^-^Ter119^-^ cells (the other cells) of the BM. *P<0.05, **P<0.01, ***P<0.001. N.D., not detected.

We have reported previously that OSM KO and OSMR KO mice exhibit mild anemic and thrombocytopenic phenotype, whereas the count of white blood cells in peripheral blood is normal [18], [19]. The hematopoietic activity in the BM of both types of KO mice evaluated by CFU assays was significantly lower than that in WT mice. Conversely, both types of KO mice showed an increase in hematopoietic activity in the spleen, compensating for the reduced hematopoiesis in the BM by the extramedullary hematopoiesis. To focus on the hematopoiesis in the BM, we performed splenectomy (Spx) and monitored the hematocrit (HCT), the volume percentage of red blood cells in peripheral blood. The Spx-treated WT mice showed a normal HCT value of ∼50% 1 week after surgery, which was maintained thereafter up to 8 weeks. By contrast, the HCT value of the Spx-treated OSM KO mice fell to ∼40% 1 week later. Interestingly, it showed no sign of recovery from anemia for 8 weeks (Fig. 1A). Erythropoietin (EPO) is a critical factor required for erythroid development and contributes to the optimal tissue oxygenation by regulating the number of peripheral erythrocytes [25]. To investigate whether the anemia in Spx-treated OSM KO mice is caused by insufficient EPO production, we measured the EPO concentration in plasma by enzyme-linked immunosorbent assay (ELISA). In WT mice, the concentration of serum EPO increased significantly by 3.9-fold after 1 week of Spx treatment (Fig. 1B), and the HCT value returned to the normal level, indicating that the kidney adequately upregulated EPO expression by sensing the hypoxic condition. Similarly, the serum EPO level of OSM KO mice increased by 2.1-fold after 1 week of Spx treatment, suggesting that the sensor for hypoxia functions normally in OSM KO mice. Despite the increase in EPO, however, the HCT values of OSM KO mice were not recovered for at least 8 weeks. Additionally, even in the steady-state condition before Spx, the serum EPO level in OSM KO mice was 2.8-fold higher than that in WT mice, suggesting that the OSM KO BM could not fully support erythropoiesis irrespective of the presence of excess EPO. These results indicate an abnormality in the BM microenvironment for hematopoietic progenitor cells in OSM KO mice.

### OSM is predominantly and constitutively expressed in the BM among IL-6 family cytokines

Because IL-6 family cytokines are known to exhibit similar activity via the shared receptor subunit gp130 [14], we examined the expression profile of IL-6 family cytokines in the BM, spleen, and liver. The expression levels of mRNA for OSM, IL-6, leukemia inhibitory factor (LIF), and ciliary neurotrophic factor (CNTF) relative to β-actin were measured by real-time reverse transcription-polymerase chain reaction (RT-PCR) (Fig. 1C). OSM was highly expressed in both the BM and spleen as previously reported, but not in the liver [26]. Compared to OSM, levels of the other cytokines in the BM were negligible, indicating that, among the IL-6 family cytokines, OSM is predominantly and constitutively expressed in the BM. Furthermore, we subdivided BM cells into blood (CD45^+^ and TER19^+^ cells) and the other cells by fluorescence-activated cell sorting (FACS) to identify the OSM-producing cells in the BM (Fig. 1D). Real-time RT-PCR revealed that hematopoietic cells were the major source of OSM regardless of inflammation, while OSM has been reported to be induced by activated macrophages and T-cells [27], [28]. Thus, the expression profile of OSM supports the idea that OSM contributes largely to the maintenance of the BM microenvironment, consistent with our previous report that the transplantation of WT BM cells into OSMR KO mice failed to recover the activity of hematopoietic progenitor cells in the recipient BM [19].

### Fatty marrow is exaggerated with age as well as by myeloablation in OSM KO mice

**Figure 2:**
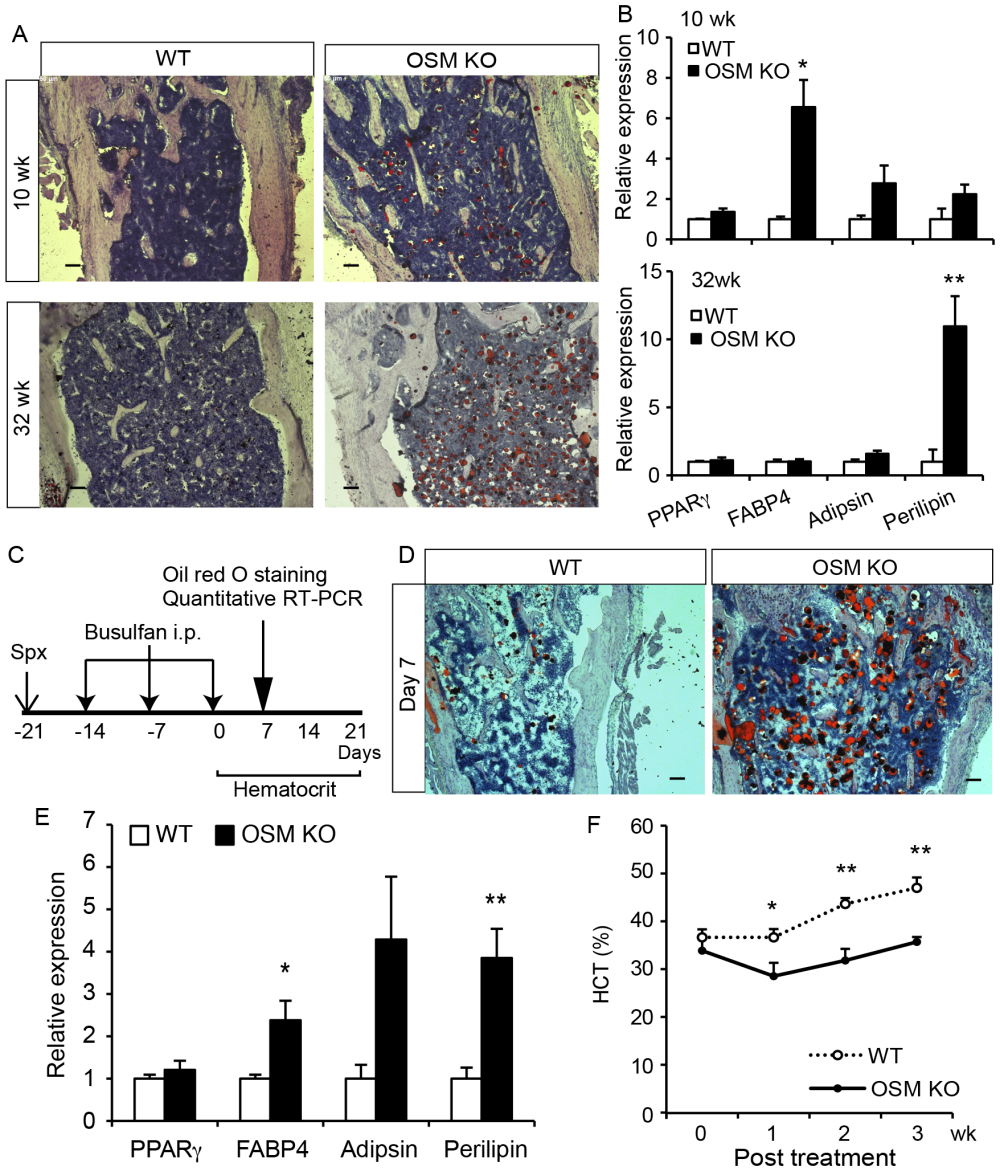
Adipogenic BM of OSM KO mice by aging and injury. (A) Oil Red O staining of femurs of young (10-week-old) and aged (32-week-old) WT and OSM KO mice (14-µm frozen sections). (B) Expression of adipogenesis-related genes by real-time RT-PCR in the BM of 10- and 32-weeks-old WT and OSM KO mice (n = 5). (C) The experimental design of chemotherapy-induced myeloablation. WT and OSM KO mice were splenectomized and then treated with an intraperitoneal injection of 20 mg/kg busulfan three times. After the final injection the hematocrit value was monitored weekly for 3 weeks. (D) Oil Red O staining of femur sections from WT and OSM KO mice 7 days after myeloablation. (E) Real-time RT-PCR analyses of the genes related to adipogenesis. The expression levels of adipsin and perilipin mRNA are shown (n = 4). (F) Hematocrit values after busulfan treatment of WT (open circle) and OSM KO mice (closed circle) (WT: 0–7 days, n = 8; 14–21 days, n = 3; OSM KO: 0–7 days, n = 5; 14–21 wks, n = 3). Data are shown as means ± S.E.M. *P<0.05, **P<0.01. Scale bars indicate 100 µm.

Because the BM adipocytes are considered to negatively affect the hematopoietic microenvironment [11], we hypothesized that the adipogenic property of OSM KO BM contributed to the impairment of hematopoietic niche. In humans and mice, it is known that the number of adipocytes in the BM increases spontaneously with age [12], [29]. To investigate whether aging accelerates the accumulation of lipid in BM of OSM KO mice, we performed Oil Red O staining of young and aged WT and OSM KO femurs. The BM of young (10-week-old) and aged (32-week-old) OSM KO mice showed mild and massive accumulation of lipid, respectively, while those of WT mice showed little staining (Fig. 2A). Consistently, the real-time RT-PCR analysis of adipogenesis-related gene expression in the BM revealed a significant increase of perilipin, which is known to be involved in the formation of lipid droplets [30], [31], in the BM of aged OSM KO mice (Fig. 2B). These results suggested that OSM is involved in the alteration of BM cellularity with ageing and that the adipogenic property and lipid accumulation in the OSM KO BM contributes to impaired hematopoietic niche in aged mice. Therefore, we analyzed the peripheral blood of 40-week-old WT and OSM KO mice (Table 1). The count of peripheral red blood cells (RBC) and platelets (PLT) in the aged OSM KO mice exhibited significant anemic and thrombocytopenic phenotypes compared to WT mice, while those phenotypes in young adult OSMR KO mice were relatively mild as reported previously [19]. In addition, the aged OSM KO mice showed marked reduction of peripheral white blood cells (WBC) number compared to WT mice, which was not observed in young adult OSMR KO [19]. These results suggested that the lack of OSM in the BM could promote fatty marrow with age, leading to pancytopenia.

**Table 1:** Hematologic analysis of the peripheral blood of 40-weeks-old mice.

	Unit	WT	OSM KO
RBC	x 10^6^/µL	9.37±0.15	7.41±0.14***
PLT	x 10^6^/µL	1.3±0.05	0.7±0.03***
HCT	%	49.8±1.53	39.6±0.64***
HGB	g/dL	14.1±0.24	12.4±0.18***
WBC	x 10^3^/µL	8.38±0.79	2.79±0.22***
Rtc	‰	32.5±1.06	27.1±1.39**
MCV	fL	52.7±0.62	53.7±0.46

RBC, red blood cells; PLT, platelets; HCT, hematocrit value; HGB, hemoglobin; WBC, white blood cells. Rtc, reticulocyte; MCV, mean corpuscular volume.

**P<0.01,

***P<0.001, WT, n = 6; OSM KO, n = 7.

Deta represent mean ± SEM.

Given that the massive accumulation of BM adipocytes in the aged OSM KO mice results from stromal cell turnover, the regenerative process of the BM microenvironment after BM lesion may elicit the adipogenesis even in young mice. Based on this idea, mice were administered with busulfan, an alkylating antineoplastic agent known to induce myeloablation of BM stromal cells as well as hematopoietic cells [32]. We injected busulfan into Spx-treated WT and OSM KO mice three times every other week to induce the reconstruction of the BM microenvironment, and then evaluated the adipogenic status in the BM 7 days after the final injection (Fig. 2C). Expectedly, marked accumulation of lipid was observed in OSM KO BM compared with WT BM by Oil Red O staining (Fig. 2D). Consistently, the expression of perilipin was strikingly upregulated in OSM KO BM during the reconstruction of the BM (Fig. 2E), indicating that myeloablation triggered severe fatty marrow in OSM KO mice. In addition, we monitored the HCT value in peripheral blood every week. While the HCT value in WT mice returned to the normal level 3 weeks after the final busulfan injection, the recovery of HCT value in OSM KO mice was blunted (Fig. 2F). These results strongly suggested that OSM plays a role in the development and maintenance of the BM microenvironment.

### OSM directly inhibits adipocytic differentiation of BM MSC *in vitro*

**Figure 3:**
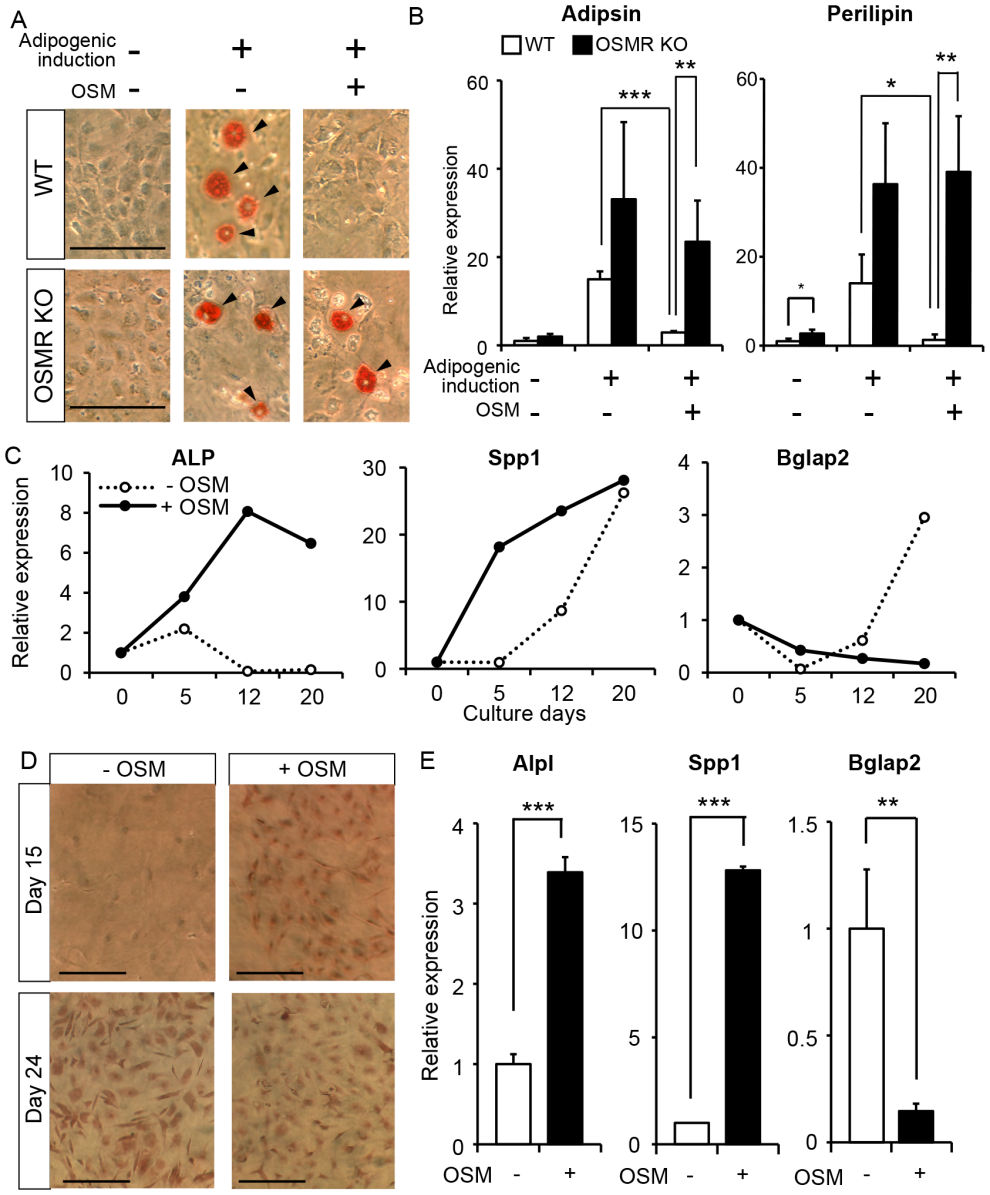
Multiple roles of OSM in differentiation of PαS cells *in vitro*. (A) Evaluation of lipid accumulation in the cells by Oil Red O staining. Adipocytic-differentiated cells are indicated by arrowheads. (B) Real-time RT-PCR analysis of genes related to adipocytic differentiation. The expression levels of adipsin, and perilipin mRNA are shown (n = 4). (C) The expression profiles of osteogenesis-related genes after induction of osteogenic differentiation. The expression levels of Alpl, Spp1 and Bglap2 were measured by real-time RT-PCR after 5, 12, and 20 days of osteogenic induction in the presence (closed circle) or absence (open circle) of 10 ng/mL OSM. (D) Alizarin Red S staining after 15 days and 24 days of induction. (E) Real-time RT-PCR analysis of Alpl, Spp1 and Bglap2 after 7 days of culture with Dex (n = 3). Data are presented as means ± S.D. *P<0.05, **P<0.01, ***P<0.001. Scale bars indicate 100 µm.

Recently, Morikawa et al. reported that a subset of MSC, so-called PαS cells in the BM, could differentiate into hematopoietic niche cells, osteoblasts and adipocytes after transplantation *in vivo* [10]. They also demonstrated the differentiation potentials of PαS cells *in vitro* into three distinct cell lineages; i.e., osteocytes, adipocytes, and chondrocytes. Therefore, we investigated whether OSM could inhibit the adipocytic differentiation of PαS cells *in vitro*. At first, we isolated PαS cells from femurs of either WT or OSMR KO mice by cell sorting, as reported previously, and named them “WT-PαS cells” and “OSMR KO-PαS cells”, respectively. As shown in S1 Fig., little difference was found between the percentages of WT-PαS cells and OSMR KO-PαS cells in BM non-hematopoietic cells. Additionally, cultured WT-PαS cells and OSMR KO-PαS cells exhibited no apparent difference in their spindle morphology in the maintenance medium culture (S1 Fig.). It is, therefore, likely that the number and characteristics of PαS cells in WT and OSMR KO BM are comparable regardless of OSM signaling *in vivo*. To examine the inhibitory effect of OSM on adipocytic differentiation of PαS cells *in vitro*, we induced adipogenesis in the presence or absence of OSM. After adipogenic induction, both WT-PαS and OSMR KO-PαS cells accumulated lipid in the absence of OSM as evaluated by Oil red O staining (Fig. 3A). By contrast, in the presence of OSM, the adipogenesis of WT-PαS was completely blocked, and the inhibitory effect was canceled in OSMR KO-PαS due to the lack of the OSM receptor. In line with that observation, the expression of fat-related genes, adipsin and perilipin were markedly downregulated by the addition of OSM in WT-PαS cell culture but not in OSMR KO-PαS cell culture (Fig. 3B). These results clearly indicated that OSM strongly inhibits the adipocytic differentiation of PαS cells *in vitro*.

### OSM accelerates osteogenic differentiation of PαS cells but suppresses their terminal differentiation *in vitro*

The finding that OSM strongly inhibited the adipocytic differentiation of PαS cells raised the possibility that OSM contributes to the hematopoietic microenvironment by biasing PαS cells towards the other type of mesenchymal stromal cell. OSM may rather enhance the osteogenic differentiation of PαS cells directly as reported in previous works using other BM MSC [22], [23]. To address the possibility, we evaluated osteogenic differentiation in WT-PαS cell culture with or without OSM. It is known that alkaline phosphatase (Alpl), secreted phosphoprotein 1 (Spp1) and bone gamma-carboxyglutamate protein 2 (Bglap2) (also known as osteopontin and osteocalcin, respectively) are expressed in osteocytic cells and that a cascade of gene induction occurs concomitantly with osteoblastic differentiation [33]. In short, Alpl is an early marker of osteogenic differentiation, but its expression decreases toward terminal differentiation. Spp1 is subsequently upregulated, and finally Bglap2 is induced and maintained in terminally differentiated osteocytes. The osteogenic differentiation of PαS induced the sequential expression of Alpl, Spp1, and Bglap2 along with osteogenesis in the absence of OSM (Fig. 3C). By contrast, the addition of OSM induced Alpl and Spp1 strongly even at the early stage of osteogenic differentiation (day 5 and day 12). Consistently, Alizarin red S staining, which reflects the extent of mineralization in the cells, demonstrated that positively stained cells appeared in the presence of OSM earlier than in the absence of OSM (Fig. 3D). These results suggested that OSM accelerates the osteogenic differentiation of PαS cells as is the case with other works using BM MSC. More intriguingly, however, we found that the sustained exposure of OSM kept the expression of Alpl at a high level while it suppressed the induction of Bglap2, a terminal differentiation marker, even after 20 days of induction (Fig. 3C). These results strongly suggested that OSM induces osteogenic differentiation of PαS cells but suppresses their terminal differentiation.

To further confirm the inhibitory effect of OSM on osteogenic terminal differentiation of PαS cells, we reexamined it in the presence of Dexamethasone (Dex), which has been reported to induce both early and late stages of osteogenic differentiation of BM stromal cells [34], [35]. Similar to the experiment without Dex, the expression levels of Alpl and Spp1 were significantly upregulated 7 days after induction in the presence of OSM, whereas that of Bglap2 was markedly suppressed (Fig. 3E). Given that immature osteolineage cells, rather than more differentiated osteoblasts, are responsible for hematopoietic microenvironment to regulate HSPC proliferation and differentiation [36], OSM may contribute to the constitution of the niche by stimulating the generation of osteolineage progenitors from PαS cells.

### OSM enhances the hematopoietic capacity of PαS-derived osteolineage cells *in vitro*

**Figure 4:**
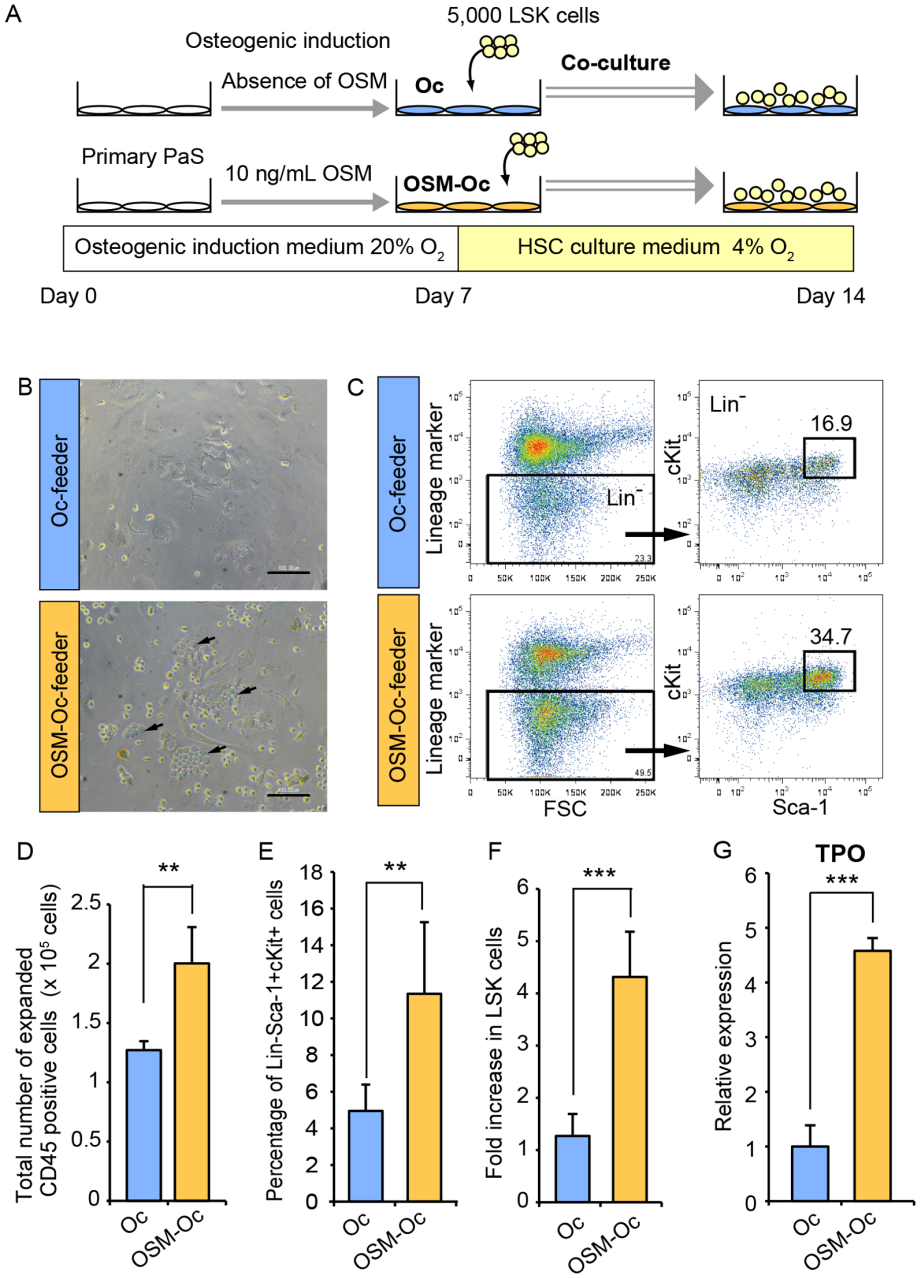
OSM enhances the capacity of PαS-derived osteoblastic cells to support hematopoisis *in vitro*. (A) The experimental schedule for the co-culture of LSK cells and the feeder layer. After the osteoblastic differentiation of PαS cells with or without OSM, LSK cells were co-cultured under 4% O_2_ conditions (balanced by N_2_). After 7 days of co-culture without OSM, cells were harvested and reanalyzed by FACS. (B) The morphology of LSK cells cultured on Oc-feeder and OSM-Oc-feeder. “Cobblestone”-like clusters (arrows) were observed in the OSM-Oc-feeder. Scale bars indicate 100 µm. (C) FACS analysis of harvested cells after 7 days of co-culture. Representative images and the percentage of the LSK cells in lineage negative CD45+ cells are shown. (D) The total numbers of expanded cells after 7 days of co-culture (n = 5). (E, F) The percentage of the LSK cells in total CD45 positive cells (E) and fold increase of LSK cells relative to input cells (F) after 7 days of co-culture are shown (n = 5). (G) Expression analysis of TPO in the Oc-feeder and OSM-Oc-feeder by real-time RT-PCR (n = 3). Data are presented as means ± S.D. **P<0.01, ***P<0.001.

To determine whether OSM can potentiate the hematopoietic capacity of osteolineage cells, we developed a co-culture system of PαS-derived osteolineage cells and Lineage-Sca1+cKit+ (LSK) cells, a fraction of HSPC in the BM (Fig. 4A). We first induced the osteogenic differentiation of PαS cells for 7 days with or without OSM, and used it as a feeder layer, which we named “Oc-feeder” and “OSM-Oc-feeder”, respectively. After wash-out of induction medium, LSK cells were added on each feeder with stem cell factor (SCF), but without OSM. After 3 days of co-culture, many “cobblestone”-like clusters were observed in the co-culture on OSM-Oc-feeder (Fig. 4B). After 7 days of co-culture, the expanded cells from each culture were harvested and reanalyzed by FACS (Fig. 4C). The total number of expanded blood cells on OSM-Oc-feeder was 1.6-fold higher than that on Oc-feeder (Fig. 4D). More importantly, FACS analysis revealed that the percentage of the LSK fraction as well as the total number of LSK cells relative to the input cells on OSM-Oc-feeder were higher than those on Oc-feeder by 2.3-fold and 3.4-fold, respectively (Fig. 4C, E and F). These results suggested that OSM-induced osteolineage cells possessed a high capacity of the *ex vivo* maintenance and expansion of HSPC. To examine the characteristic difference between Oc-feeder and OSM-Oc-feeder, the expression level of Thrombopoietin (TPO), a critical factor for hematopoiesis, was analyzed. Real-time RT-PCR revealed that the expression of TPO in the OSM-Oc-feeder was significantly higher than the Oc-feeder by 4.6-fold, which may account for a part of niche functions (Fig. 4G), although we cannot exclude the possibility that the other cytokines than TPO or the direct interaction between LSK and the feeder layer might be responsible for high capacity of hematopoiesis. Taken together, these results suggested that OSM plays a role in the development of the favorable microenvironment for HSPC by preventing PαS cells from osteogenic maturation as well as adipogenesis.

### OSM administration facilitates the reconstitution of the BM microenvironment in lethally irradiated mice

**Figure 5:**
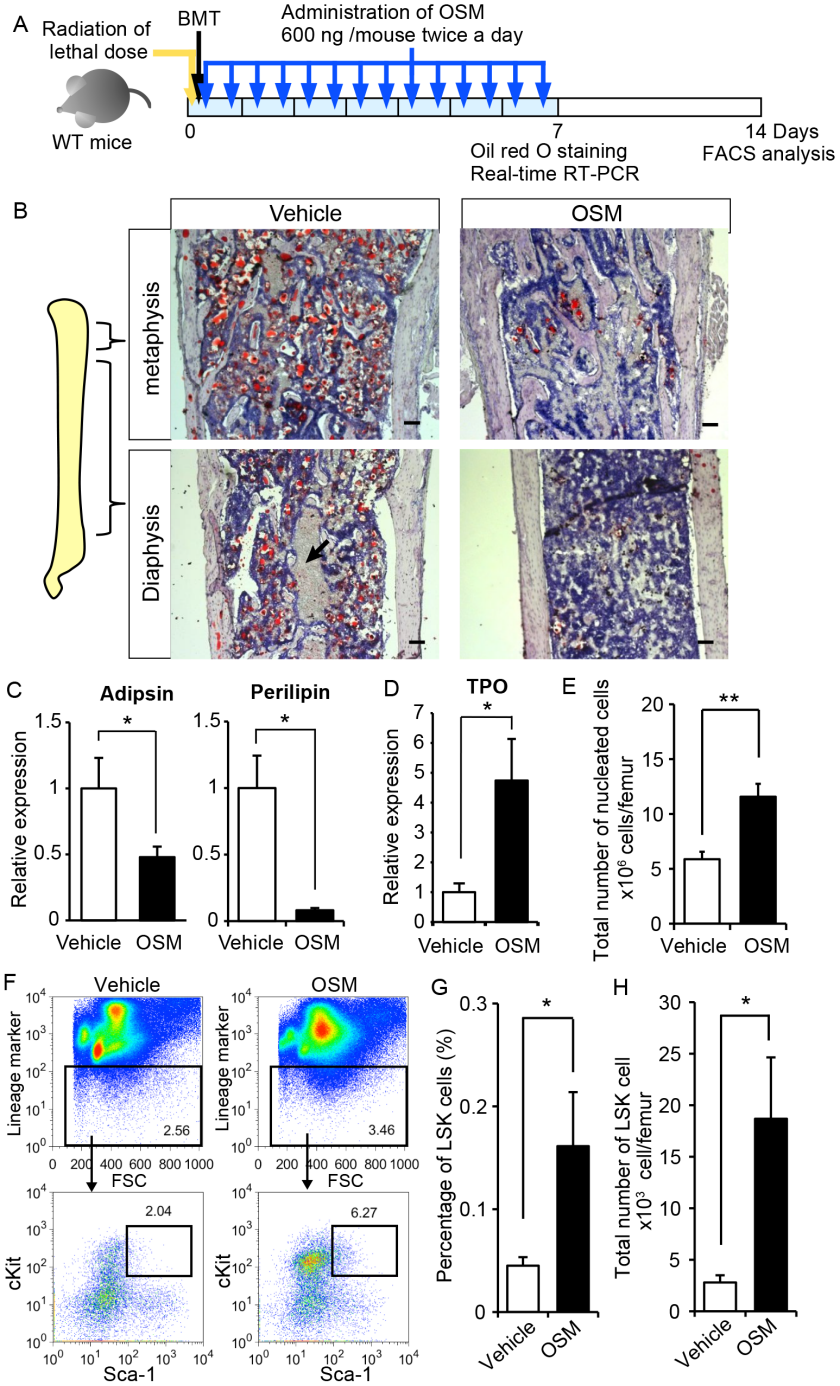
OSM suppresses fatty marrow and enhances the recovery of BM microenvironment after irradiation *in vivo*. (A) The experimental schedule for irradiation and OSM administrations. BM cells were transplanted into lethally irradiated WT mice by tail vein injection. A dose of 600 ng OSM per mouse was injected intraperitoneally twice a day for 7 days. (B) Oil red O staining of femur sections from PBS-treated mice (Vehicle) and OSM-treated mice. Arrow shows the open area occupied by erythrocytes. (C) Real-time RT-PCR analysis of genes related to adipocytic differentiation. The expression levels of adipsin and perilipin mRNA are shown (n = 4-5 per group). (D) Expression analysis of TPO in the BM by real-time RT-PCR (Vehicle, n = 4; OSM-treated mice, n = 5). (E) The total number of BM cells per a femur after 14 days of BMT. (F) FACS analysis of BM cells in vehicle-treated and OSM-treated mice. Representative images and the percentage of LSK cells in BM cells are shown. (G) The percentage of LSK cell in BM cells. (H) The LSK number in the BM per a femur. (Vehicle, n = 6; OSM-treated mice, n = 7). Data are shown as means ± S.E.M. *P<0.05, **P<0.01, ***P<0.001.Scale bars represent 100 µm.

Because OSM is predominantly and constitutively expressed in hematopoietic cells of the BM (Fig. 1C and D), the myelosuppression by lethal irradiation is supposed to cause an insufficient supply of OSM even in the WT BM. To assess the therapeutic advantage of OSM administration for BM microenvironment recovery after myeloablation, WT mice were lethally irradiated and transplanted with BM cells (BMT). After BMT, recombinant OSM was intravenously administered at a dose of 600 ng per a mouse twice a day for a week and then the BM reconstitution was evaluated (Fig. 5A). Oil Red O staining revealed that OSM treatment markedly suppressed the accumulation of lipids in whole BM compared to vehicle injection, indicating that the administration of OSM inhibits the BM adipogenesis *in vivo* (Fig. 5B). Moreover, OSM-treated BM was filled with nucleated hematopoietic progenitor cells whereas vehicle-treated BM displayed many open areas occupied by enucleated red blood cells (Fig. 5B, arrow). Real-time RT-PCR revealed that the expressions of adipsin and perilipin in the BM of OSM-treated mice were strongly suppressed by 0.48-fold and 0.08-fold compared to the vehicle-treated BM, respectively (Fig. 5C). In contrast, the expression of TPO was 4.7-fold increased in the BM of OSM-treated mice, consistent with the *in vitro* data described above (Fig. 5D and Fig. 4G). These data indicate that the administration of OSM is useful for inhibiting the adipogenesis during the regeneration of BM microenvironment, which would contribute to the recovery of hematopoiesis.

### OSM administration enhances the recovery of BM hematopoiesis after lethal irradiation

To evaluate the effect of OSM administration on the hematopoietic recovery in the BM, the nucleated cells were harvested from vehicle- and OSM-treated femurs after 14 days of BMT and analyzed by FACS. The total number of BM nucleated cells per a femur of OSM-treated mouse was 2.0-fold higher than that of vehicle-treated mouse (Fig. 5E). FACS analysis demonstrated that the percentage of the LSK fraction in total nucleated cells as well as the total number of LSK cells per a femur of OSM-treated mouse were higher than those of vehicle-treated mouse by 4.1-fold and 6.7-fold, respectively (Fig. 5F, G, and H). These results strongly suggested that *in vivo* administration of OSM after lethal irradiation has a beneficial effect on the rapid recovery of hematopoietic microenvironment in the BM.

**Figure 6:**
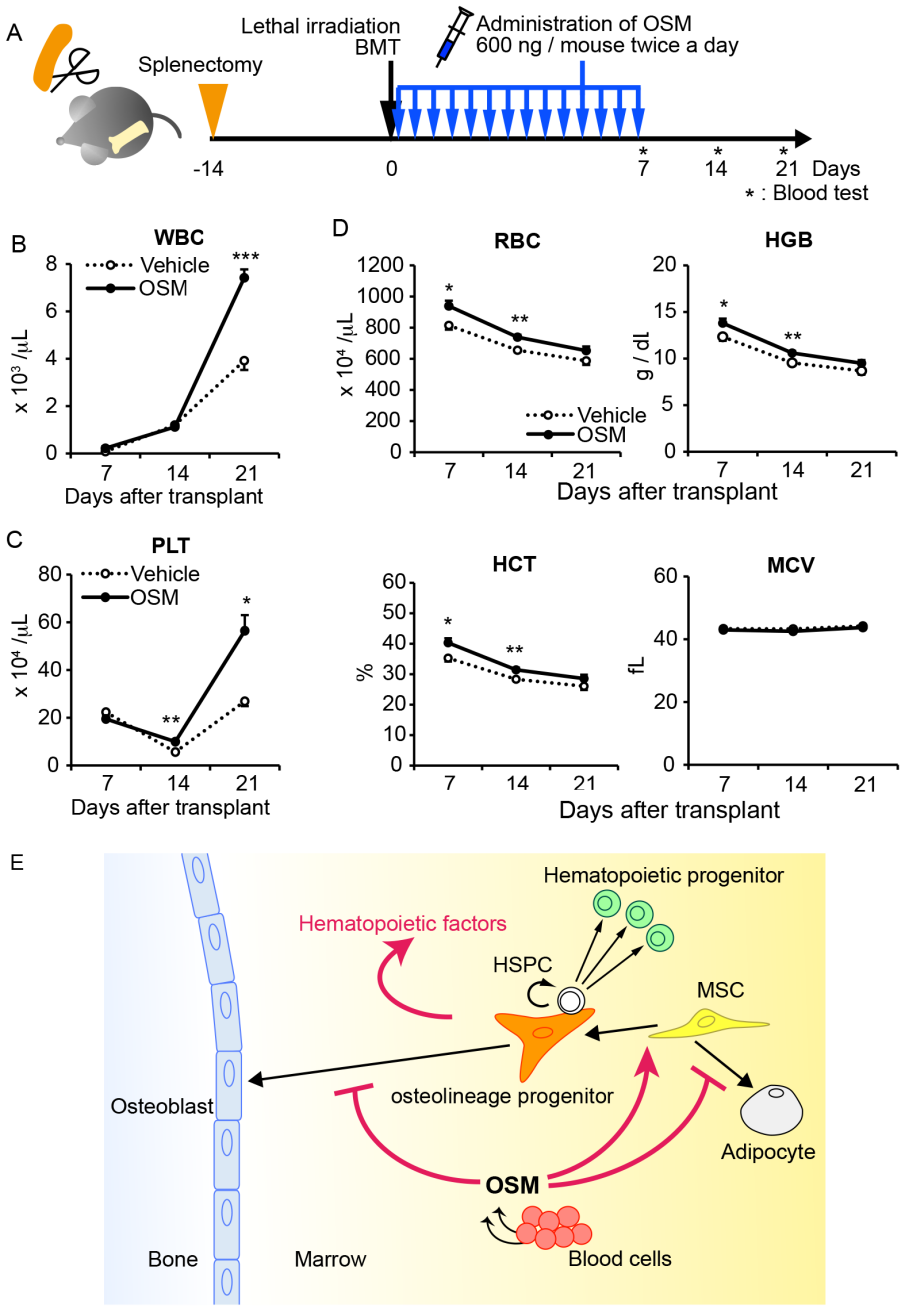
OSM enhances the recovery of BM hematopoiesis after irradiation *in vivo*. (A) The experimental schedule for splenectomy, irradiation and OSM administrations. WT mice were irradiated at lethal dose after 14 days of splenectomy and then a dose of 600 ng OSM per mouse was injected intraperitoneally twice a day for 7 days. Blood samples were harvested from tail vein and analyzed by automated counter every 7 days. (B-D) Hematologic analyses of peripheral blood after BMT. While blood cell count (WBC) (B) platelet cell count (PLT) (C) were measured by an automated counter. (D) Red blood cell count (RBC), mean corpuscular volume (MCV), hemoglobin content (HGB), and hematocrit values (HCT) are shown. (E) Model of multiple regulatory roles of OSM in the BM stromal cell differentiation and hematopoietic microenvironment. Data are shown as means ± S.E.M. (n = 5 per group). *P<0.05, **P<0.01, ***P<0.001.

To further focus on the contribution of BM recovery to peripheral blood, we performed similar BMT experiments for Spx-treated WT mice and monitored the peripheral blood every 7 days after BMT (Fig. 6A). While the recovery of peripheral WBC and PLT was blunted in vehicle-treated mice, OSM administration elicited rapid recovery of WBC and PLT, and their counts were significantly higher than vehicle-treated mice after 21 days of BMT by 1.9- and 2.1-fold, respectively (Fig. 6B and C). Similarly, the values of RBC, HCT and hemoglobin (HGB) in OSM-treated mice were consistently higher than those of vehicle-treated mice (Fig. 6D).

Furthermore, we examined the hematopoietic recovery in Spx-treated OSM KO mice after lethal irradiation with WT BM transplantation. Although the administration of OSM was expected to be more effective in OSM-deficient BM environment, we could not find its clear advantage in the recovery of peripheral blood nor BM LSK number (S2 Fig.). Because the BM of OSM KO mouse is originally adipogenic, the pre-existing lipids in the KO BM may be less affected by OSM administration while the renewed adipogenesis in WT BM after irradiation is effectively blocked. Altogether, these results indicated that OSM administration was beneficial to suppress the adipogenesis after myeloablation, leading to the recovery of BM microenvironment as well as hematopoiesis.

## Discussion

In the present study, we focused on the effect of OSM on PαS cell because the cell has been reported to differentiate into hematopoietic niche cells, osteoblasts, and adipocytes after *in vivo* transplantation [10]. We demonstrated that OSM exhibits distinct biological activities against adipogenesis and osteogenesis of PαS cells. We previously reported the inhibitory effect of OSM on the adipocytic differentiation of 3T3-L1, a preadipocyte line [20] and that both OSM KO and OSMR KO mice displayed an anemic phenotype accompanied by the reduction of hematopoietic activity in the BM [18], [19]. Here, we showed that OSM is expressed constitutively and abundantly in the BM, a unique feature of OSM among the IL-6 family cytokines. Moreover, the adipogenesis in OSM KO BM was accelerated with age as well as after myeloablation. These results strongly suggest that OSM plays a critical role in the development and/or maintenance of the BM microenvironment. Considering that adipogenic change occurs drastically and extensively in the BM after irradiation and that PαS cells are relatively rare in marrow cavities, OSM may contribute to hematopoietic microenvironment by affecting the other type of BM MSC as well as PαS cells. Adipocytes in the BM are considered to be a negative regulator of the hematopoietic microenvironment, raising the possibility that the administration of a chemical inhibitor of adipogenesis might enhance marrow engraftment and hematopoietic recovery after irradiation by antagonizing BM adipogenesis [11]. Therefore, the regulatory mechanisms underlying BM adipogenesis in the steady state as well as under disease conditions would help us to understand the hematopoietic microenvironment and develop novel therapeutic strategies. Because impaired hematopoiesis in the BM is often compensated by extramedullary hematopoiesis in the spleen, fine regulatory mechanisms in BM hematopoiesis may be masked by the compensatory hematopoiesis. Therefore, we utilized Spx-treated mice to focus on the BM hematopoiesis. In BMT model, one of the myeloablation models, Sxp-treated WT mice markedly showed the rapid recovery of peripheral WBC and PLT by OSM administration, suggesting that anti-adipogenic effect of OSM is useful for the recovery of hematopoietic microvenvironment in the BM. Unexpectedly, OSM administration into irradiated OSM KO mice did not exhibit enough effect on the recovery of BM hematopoiesis after irradiation. Considering that OSM effectively blocks an early step of adipocytic differentiation [20], it may need more time to replace the pre-existing adipocytes in the OSM KO BM. Therefore, the span and dose of OSM administration need further consideration to improve the BM microenvironment of OSM KO mouse.

Interestingly, OSM KO mice showed some characteristics similar to the diagnostics of AA; i.e., fatty marrow, high serum EPO concentration, a high frequency in aged individuals, and anemia. For many patients with severe AA, transplantation of BM or cord blood cells is the preferred standard treatment [37], [38]. Transplantation is thought to replace the abnormal hematopoietic progenitor cells in the BM with normal HSPC because hematopoietic progenitors themselves are of pathogenic importance. Our data suggest that defective regulatory molecules for the BM microenvironment could also be linked to the pathogenesis of AA. Notably, OSM is a potentially promising agent for the protection of fatty marrow, although further investigation will be required to clarify the relationship between AA and OSM expression in the BM.

BM contains various cell types involved in the formation of the hematopoietic microenvironment [1], [39], [40]. Previous studies have revealed that various stromal cells, such as osteoblasts [2], [3], osteocytes [41], perivascular Nestin-expressing MSC [42], CXCL12-expressing cells [43]–[46], and BM sinusoidal endothelial cells [5], [7], [47], contribute to the formation of the BM hematopoietic niche. Among the multiple cell types in the BM, the osteoblast is the first to be identified as a functional niche cell, although several lines of evidence suggest that the role of osteoblasts in HSC regulation is not as it was initially foreseen [48]. It is likely that the osteoblasts constituting the HSC niche are relatively immature, because CD146^+^ osteoprogenitors, but not their differentiated osteoblastic progeny, express Angiopoietin-1, a pivotal regulator both of vascular remodeling and of the HSC niche [49]. Moreover, osteolineage cells are also known to express some secreted proteins required for hematopoiesis; e.g., TPO, which enhances LT-HSC quiescence, and Spp1, an extracellular matrix molecule, which enhances the quiescence of primitive HSC through its binding to integrin β1 [50], [51]. We demonstrated that OSM enhanced the expression of TPO and Spp1 in osteolineage cells derived from PαS cells, and suppressed the expression of Bglap2, a terminal differentiation marker of osteocytic cells, suggesting that OSM also contributes to the supply of stromal cells constituting hematopoietic niche. In fact, OSM-treated osteolineage cells showed marked potentials for maintenance and expansion of the LSK fraction as feeder cells in the co-culture system. These findings may account for the finding in our previous report that the number of hematopoietic progenitor cells in peripheral blood was increased in OSM KO mice, presumably due to the impaired ability of the BM niche to harbor HSPC.

In conclusion, our findings demonstrate that OSM plays multiple regulatory roles in BM stromal cell differentiation and is required to maintain the BM microenvironment for hematopoiesis (Fig. 6E). OSM is a promising therapeutic target for alleviating the BM diseases with fatty marrow as well as myelosuppression after chemotherapy or irradiation. Further characterization of the osteolineage progenitors responsible for the OSM effect would help us to understand the regulatory mechanisms of BM microenvironment for hematopoiesis and to identify novel niche factors for HSPC.

## Supporting Information

S1 Fig **Comparison between PαS cells derived from WT and OSMR KO mice.** (A) WT-PαS cells and OSMR KO-PαS cells were sorted as CD45^-^ TER119^-^ and Sca-1^+^ PDGFRα^+^ population by FACS. (B) The morphology of primary WT-PαS cells and OSMR KO-PαS cells after 7 days of culture. Bars indicate 100 µm.
(TIF)

S2 Fig **Analysis of the recovery of peripheral blood cells and HSPC in the BM in Spx-treated OSM KO mice.** (A) The experimental schedule for splenectomy, irradiation and OSM administrations using OSM KO mice. OSM KO mice were irradiated at lethal dose after 14 days of splenectomy and then a dose of 600 ng OSM per mouse was injected intraperitoneally twice a day for 7 days. Blood samples were harvested from tail vein and analyzed by automated counter every 7 days. (B) Hematologic analyses of peripheral blood after BMT. The transition of while blood cell count (WBC), platelet cell count (PLT) and red blood cell count (RBC) in vehicle-treated and OSM-treated mice are shown. (C) The total number of BM cells per a femur, the percentage of LSK cell in BM cells, and the LSK number in the BM per a femur after 21 days of BMT are shown. (Vehicle, n = 4; OSM-treated mice, n = 5). Data are shown as means ± S.E.M.
(TIF)

S1 Table **Primer sequences for real-time RT-PCR.** All primer sequences used in this study are shown.
(DOCX)
