# Supplementary figures and images for: Oncostatin M Maintains the Hematopoietic Microenvironment in the Bone Marrow by Modulating Adipogenesis and Osteogenesis
Source: PLoS One. 2014 Dec 31;9(12):e116209. doi: 10.1371/journal.pone.0116209 (PMC4281151; doi:10.1371/journal.pone.0116209)

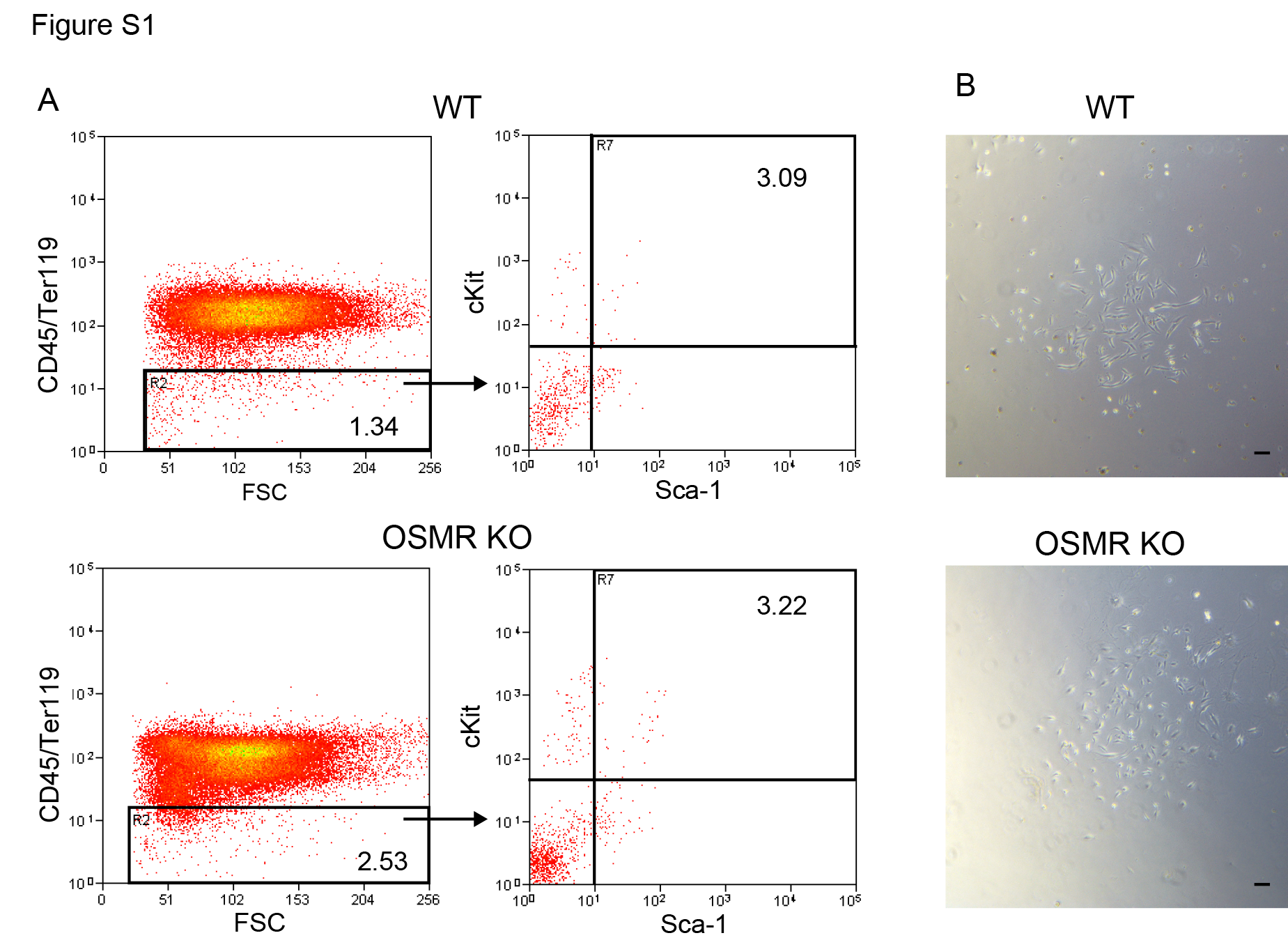

Supplement: S1 Fig — Comparison between PαS cells derived from WT and OSMR KO mice. (A) WT-PαS cells and OSMR KO-PαS cells were sorted as CD45- TER119- and Sca-1+ PDGFRα+ population by FACS. (B) The morphology of primary WT-PαS cells and OSMR KO-PαS cells after 7 days of culture. Bars indicate 100 µm. (TIF) [file pone.0116209.s001.tif]

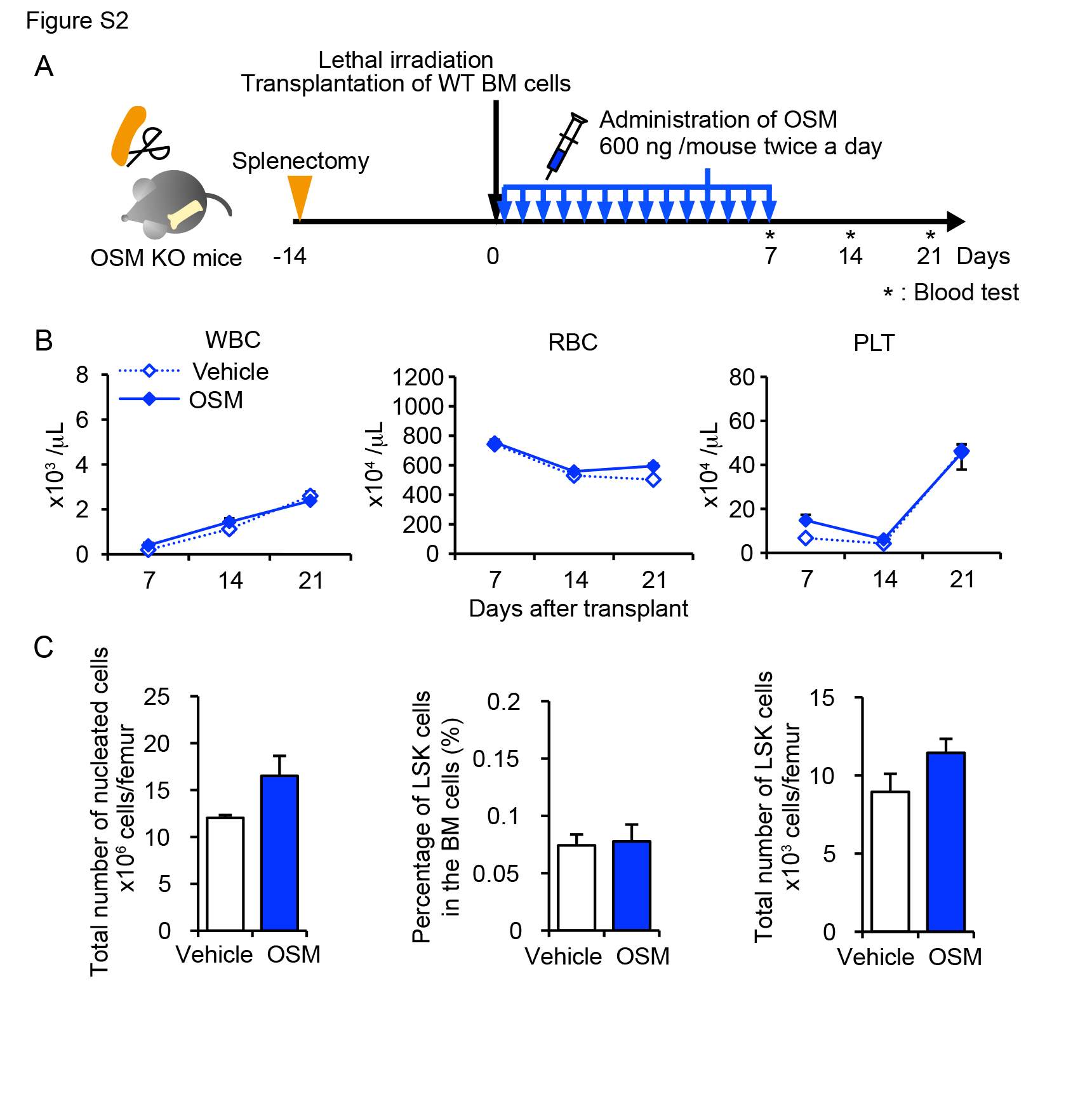

Supplement: S2 Fig — Analysis of the recovery of peripheral blood cells and HSPC in the BM in Spx-treated OSM KO mice. (A) The experimental schedule for splenectomy, irradiation and OSM administrations using OSM KO mice. OSM KO mice were irradiated at lethal dose after 14 days of splenectomy and then a dose of 600 ng OSM per mouse was injected intraperitoneally twice a day for 7 days. Blood samples were harvested from tail vein and analyzed by automated counter every 7 days. (B) Hematologic analyses of peripheral blood after BMT. The transition of while blood cell count (WBC), platelet cell count (PLT) and red blood cell count (RBC) in vehicle-treated and OSM-treated mice are shown. (C) The total number of BM cells per a femur, the percentage of LSK cell in BM cells, and the LSK number in the BM per a femur after 21 days of BMT are shown. (Vehicle, n = 4; OSM-treated mice, n = 5). Data are shown as means ± S.E.M. (TIF) [file pone.0116209.s002.tif]
