# Supplementary material for: Oncostatin M Maintains the Hematopoietic Microenvironment in the Bone Marrow by Modulating Adipogenesis and Osteogenesis
Source: PLoS One. 2014 Dec 31;9(12):e116209. doi: 10.1371/journal.pone.0116209 (PMC4281151; doi:10.1371/journal.pone.0116209)
Supplement: S1 Table — Primer sequences for real-time RT-PCR. All primer sequences used in this study are shown. (DOCX) [file pone.0116209.s003.docx]

Table S1. Primer sequences for real-time RT-PCR

| OSM | Forward | 5’-tgctccaactcttcctctcag-3’ |
| --- | --- | --- |
|  | Reverse | 5’-caggttttggaggcggata-3’ |
| IL-6 | Forward | 5’-gctaccaaactggatataatcagga-3’ |
|  | Reverse | 5’-ccaggtagctatggtactccagaa-3’ |
| LIF | Forward | 5’-aacgggacagagaagaccaa-3’ |
|  | Reverse | 5’-agggaggcgctcaggtat-3’ |
| CNTF | Forward | 5’-tgattcccaggcacaaaatc-3’ |
|  | Reverse | 5’-ccctgcctgaatcagaggt-3’ |
| PPARg | Forward | 5’-caagccctttaccacagttga-3’ |
|  | Reverse | 5’-cagctcttgtgaatggaatgtc-3’ |
| FABP4 | Forward | 5’-ggatggaaagtcgaccacaa-3’ |
|  | Reverse | 5’-tggaagtcacgcctttcata-3’ |
| Adipsin | Forward | 5’-ctgggagcggctgtatgt-3’ |
|  | Reverse | 5’-cacggaagccatgtaggg-3’ |
| Perilipin | Forward | 5’-ggatggagacctccctgag-3’ |
|  | Reverse | 5’-ctcacaggtcccgctcac-3’ |
| Alpl | Forward | 5’-aatgaggtcacatccatcctg-3’ |
|  | Reverse | 5’-cacccgagtggtagtcacaa-3’ |
| Spp1 | Forward | 5’-cccggtgaaagtgactgatt-3’ |
|  | Reverse | 5’-ttcttcagaggacacagcattc-3’ |
| Bglap2 | Forward | 5’-agactccggcgctacctt-3’ |
|  | Reverse | 5’-ctcgtcacaagcagggttaag-3’ |
| TPO | Forward | 5’-tagcctgggagaatggaaaa-3’ |
|  | Reverse | 5’-tccctccagtagaagggaca-3’ |
